# Massachusetts Veterinary Association

**Published:** 1895-02

**Authors:** John M. Parker

**Affiliations:** Secretary


### MASSACHUSETTS VETERINARY ASSOCIATION.

THE regular monthly meeting was held at 19 Boylston Place, on Wednesday, November 28, 1894, at 7.30 P.M.

The members present were Drs. Becket, Blackwood, Burr, Howard, Labau, Marshall, Osgood, Paige, Parker, Peters, Rogers, Winchester, Winslow, Lewis, and Stickney. The President, Dr. Burr, occupied the chair.

The minutes of the last meeting having been read and adopted, the resignation of Dr. Lee was considered. Dr. Parker moved that his resignation be accepted; seconded by Dr. Winchester; carried.

The report of the Committee on Tuberculosis was then presented by the Secretary. The sections were taken up one by one and considered, and finally the report was adopted. The report is as follows:

"Realizing the importance of preventing the extension and continuance of bovine tuberculosis among our dairy herds, the Massachusetts Veterinary Association has prepared the following brochure in the hope that it may assist in spreading information on the prevention and eradicatlon of this disease.

"In referring to tuberculosis the following questions are often asked by stock owners: First, how shall the occurrence of tuberculosis be prevented in a healthy herd; and second, how shall tuberculosis be eradicated from a herd that is already diseased.

"In considering these questions it should be borne in mind that while it is a fact that, no matter how unhealthy the surroundings, bovine tuberculosis cannot exist without the presence of the bacillus, yet it is equally a fact that the germ requires a suitable soil for its development, and that a favorable condition of the body for the development of tuberculosis frequently rseults from hereditary predisposition, unsanitary surroundings, and the injudicious management to which dairy cattle are so often subjected. It follows, then, that anything that tends to undermine the health of dairy stock should be avoided and a continual effort made to strengthen and build up the constitution of the dairy cow.

"The following recommendations are made to stock owners to prevent the occurrence of tuberculosis in a healthy herd:

"1. As far as possible owners should raise their own stock and endeavor to improve the constitution of the herd by breeding only from animals that are strong constitutionally and known to be free from any tuberculous taint.

"2. When practicable, all farmers should own a bull. They should restrict its use to their own cows and not allow it to come in contact with other stock.

"3. Allow no strange animal to come in contact with the herd without first making sure by tuberculin test (which is now recognized to be the only practical method of diagnosis) that it is free from disease.

"4. Never buy from infected or suspicious herds.

"5 Never purchase a cow with a cough or abnormal breathing, lumpy or diseased udder, swollen joints, or with a tendency to scour or bloat.

"6. Overcrowding in barns should be avoided. Provide as much air space as possible, allowing at least 1000 cubic feet for each animal.

"7. Pure air and abundant sunlight are essential to the preservation of health in animals. Windows hinged at the bottom and dropped slightly inward at the top may be utilized for light and ventilation. In this way the air is directed upward, thereby preventing a current of cold air on the cattle.

"8. In fair weather cattle should be in the open air as much as possible.

"9. All barns should be kept as clean as possible, they should be sprinkled before bring swept, and in consequence of the irritating and infectious character of the dust of stables in which tuberculous animals have been kept, sweeping should always be done while the cattle are in the yard.

"10. In consequence of the danger to cattle from consumptives expectorating in and around the barns, no consumptive person should be allowed to have charge or come in contact with dairy cattle.

"11. Do not keep manure in the cellar. Better have no cellar; but where one exists, it should be well drained, well lighted, and well ventilated.

"12. Manure should be frequently removed from the neighborhood of barns.

"13. The barnyard and its surroundings should be well drained and free from standing water and filth.

"14. Early breeding, late and continuous breeding, as well as excessive and injudicious feeding and milking, are all frequent predisposing causes, and should be avoided.

"With reference to the eradication of the disease in herds already affected, it is recommended that a thorough examination of the herd be made, using tuberculin test. All animals found diseased should be slaughtered and the remaining animals retested at intervals. The thorough disinfection and renovation of all infected barns is imperative, and good drainage, light, and ventitation should be secured. Where these conditions cannot be obtained, it would in many cases be more economical and satisfactory to build new stables, always observing the recommendations suggested for healthy herds."

Dr. Winchester moved that the report be published in the agricultural weeklies and other papers throughout the State, and that copies be sent to Secretary Sessions for distribution; seconded by Dr. Osgood, and carried.

Dr. Winchester also moved that a vote of thanks be given the committee for their services covering nearly twelve months. The committee were then discharged with thanks.

The names of Drs. Hamilton and Pope were then voted on, and they were unanimously elected members of the association.

The gift of Messrs. Hemenway and Morgan to Harvard Veterinary College was discussed, and Dr. Peters introduced the following motion: *Whereas*, The Massachusetts Veterinary Association have expressed from time to time their entire disapprobation of the subscription plan as carried on by the Harvard Veterinary School since its inception, *Resolved*, that as the school has been enabled to abolish this system through the kindness of Messrs. Augustus Hemenway and E. D. Morgan, this association expresses its approval of the same as a step in advance, and that we tender our thanks to the friends of the veterinary profession who have subscribed an amount sufficient to provide for the abolishment of this evil. After some further discussion the motion was carried unanimously.

An interesting discussion then arose as to variations in normal temperature and thermometrical readings in using tuberculin. Drs. Peters, Paige, Osgood, Winchester, and others took part. It seemed to be the general opinion that tuberculin should be used only with the greatest caution. Dr. Osgood's method was to give little hay or food while the readings were being taken; he expressed himself as being more afraid of a rise of temperature after feed, ing than a lowering of temperature after watering.

Before the meeting adjourned Dr. Parker said he thought a committee on legislation should be appointed by the association to look after the interests of the profession, and to report to the association on the proposed legislation the coming winter. The consideration of the matter was postponed till next meeting, and, there being no further business, the meeting adjourned.

JOHN M. PARKER,
Secretary.

